# Are physical symptoms among survivors of a disaster presented to the general practitioner? A comparison between self-reports and GP data

**DOI:** 10.1186/1472-6963-7-150

**Published:** 2007-09-21

**Authors:** Bellis van den Berg, C Joris Yzermans, Peter G van der Velden, Rebecca K Stellato, Erik Lebret, Linda Grievink

**Affiliations:** 1 National Institute for Public Health and the Environment (RIVM), Bilthoven, The Netherlands; 2 Institute of Risk Assessment Sciences (IRAS), Utrecht University, Utrecht, The Netherlands; 3 Netherlands Institute for Health Services Research (NIVEL), Utrecht, The Netherlands; 4 Institute for Psychotrauma (IvP), Zaltbommel, The Netherlands; 5 Centre for Biostatistics, Utrecht University, Utrecht, The Netherlands

## Abstract

**Background:**

Most studies examining medically unexplained symptoms (MUS) have been performed in primary or secondary care and have examined symptoms for which patients sought medical attention. Disasters are often described as precipitating factors for MUS. However, health consequences of disasters are typically measured by means of questionnaires, and it is not known whether these self-reported physical symptoms are presented to the GP. It is also not known if the self-reported symptoms are related to a medical disorder or if they remain medically unexplained. In the present study, three research questions were addressed. Firstly, were self-reported symptoms among survivors presented to the GP? Secondly, were the symptoms presented to the GP associated with a high level of functional impairment and distress? Thirdly, what was the GP's clinical judgment of the presented symptoms, i.e. were the symptoms related to a medical diagnosis or could they be labeled MUS?

**Methods:**

Survivors of a man-made disaster (N = 887) completed a questionnaire 3 weeks (T1) and 18 months (T2) post-disaster. This longitudinal health survey was combined with an ongoing surveillance program of health problems registered by GPs.

**Results:**

The majority of self-reported symptoms was not presented to the GP and survivors were most likely to present persistent symptoms to the GP. For example, survivors with stomachache at both T1 and T2 were more likely to report stomachache to their GP (28%) than survivors with stomachache at only T1 (6%) or only T2 (13%). Presentation of individual symptoms to the GP was not consistently associated with functional impairment and distress. 56 – 91% of symptoms were labeled as MUS after clinical examination.

**Conclusion:**

These results indicate that the majority of self-reported symptoms among survivors of a disaster are not presented to the GP and that the decision to consult with a GP for an individual symptom is not dependent on the level of impairment and distress. Also, self-reported physical symptoms such as headache, back pain and shortness of breath are likely to remain medically unexplained after the clinical judgment of a GP.

## Background

Symptoms such as fatigue, stomachache, and headache are very common [1]; an estimated 80% of the general population experiences at least one such symptom in any given month [2,3]. Primary care studies have also shown that, when presented to the general practitioner (GP), at least one-third of these symptoms cannot be related to a medical disease after clinical judgment, and can be labeled as medically unexplained symptoms (MUS) [1,4]. MUS are strongly associated with a high level of functional impairment and psychological problems, such as depression and anxiety [4,5]. Because MUS are defined as physical symptoms that have no clinically determined pathogenesis after an appropriately thorough diagnostic evaluation [6], most studies have examined MUS among primary or secondary care patients.

Although these symptoms are a major reason to seek medical care [3,4], the majority of symptoms are transient and not considered severe enough to seek medical attention and are therefore not presented to the GP [2,3]. This results in a large reservoir of symptoms in the general population that has not been studied in primary and secondary care studies [7]. Engel and Katon have described MUS as a four-part process [6]. An individual must first experience the symptoms. Cognition, related to how individuals think about the symptoms, follows. This second step includes beliefs about the cause of symptoms and assignment of medical importance. In the third step, an individual seeks medical care for the symptoms, an act that is mediated by the belief in the symptom's significance. The judgment of the clinician concerning whether the symptoms can be explained by a medical disorder comprises the fourth step. It can be argued that the symptoms reported in a questionnaire represent the symptoms in step one and that the symptoms presented to the GP are those from steps three and four.

Traumatic events, such as disasters, have been described as important precipitating factors for MUS [8-11]. In these studies after disasters, questionnaires were used to examine symptoms [11]. Although questionnaires are very useful in the study of health problems in epidemiologic studies, they do not provide insight into the steps two through four in the four-part process described by Engel and Katon. For that reason, it is unknown whether or not survivors of disasters are more likely to seek medical care for their health problems than individuals in the general population. Since survivors of disasters might have different cognitions about their symptoms, their health care utilization for symptoms may be different from that of the general population.

In the present study among survivors of a man-made disaster, we compared self-reported symptoms with symptoms registered in the electronic medical systems of GPs. Three research questions were addressed. Firstly, were self-reported symptoms among survivors presented to the GP? Secondly, were the symptoms presented to the GP associated with a high level of functional impairment and distress? Thirdly, what was the GP's clinical judgment of the presented symptoms, i.e. were the symptoms related to a medical diagnosis or could they be labeled MUS?

## Methods

### Study design and participants

We combined two data collection methods: a longitudinal health survey among survivors, using self-administered questionnaires, and an ongoing surveillance program in which health problems were registered by GPs in the electronic medical records (EMRs) of survivors.

The first health survey was performed 3 weeks (T1) after the explosion of a fireworks depot in a residential area in the city of Enschede, the Netherlands (13 May 2000). As a result of the explosions and the subsequent fire, 23 people were killed, over 900 people were injured and approximately 1200 people were forced to relocate because their houses were severely damaged or destroyed. All residents who were living in the disaster area (as designated by the municipality) were considered survivors. All survivors of the disaster were invited to participate in the health survey by means of announcements in the local media and letters. On the basis of the list of addresses at the municipal registry office, it was confirmed that the total affected group living in the disaster area consisted of 4456 adult residents. In total, 1567 affected residents (response ≅ 30%) completed a questionnaire at T1. Approximately 18 months after the disaster, in November 2001, a second survey (T2) was conducted. Affected residents (i.e. survivors) who had completed a questionnaire at T1 and who had given informed consent for future contact received an announcement letter (N = 1551). In total, 1116 survivors (response 72.0%) participated at T2. The study protocol of this longitudinal study was approved by the Medical Ethical Testing Committee (TNO-Leiden-The Netherlands). Details of the study population, non-response and procedures of the surveys have been described elsewhere [12-15].

In addition to the health survey, all GPs in Enschede were invited to participate in the surveillance program. Dutch citizens are required to be registered at the list of one GP whom they consult for their health problems, and who serves as the gatekeeper for secondary care. In total, 44 out of 60 GPs agreed to participate (73%). Of the non-participating GPs, nine did not have affected residents in their practice. Patients were informed about the participation of their GP in the surveillance program, and nobody denied access to their medical information.

### Measures

#### Self-reported Symptoms

Symptoms were measured at T1 and T2 by the 13-item VOEG scale (Questionnaire into Subjective Health Complaints), a validated questionnaire which has often been used for studies in the Dutch population [16]. The items of this scale ask respondents whether (yes/no) they regularly suffer from symptoms such as headache, back pain, and fatigue (table 1). To make the symptoms compatible with the International Classification of Primary Care (ICPC) [17], the classification system used by the GPs, different stomach and fatigue items were grouped. In addition, listlessness and tingling in arms and legs were excluded because they were not compatible with any of the ICPC codes.

**Table 1:** Demographic characteristics of participants at T1 and T2 and nonparticipants in the health survey for the entire cohort of residents affected by the fireworks disaster in Enschede

**Characteristic**	**Participated at T1 & TT2 of the health survey **N = 1116	**Did not participate in the health survey **N = 3285
Female gender %	54.8	45.7 ***
Age		
18 – 24	10.0	22.8 ***
25 – 44	45.0	39.9
45 – 64	34.4	22.6
≥ 65	10.6	14.7
Family situation %		
Living alone	33.1	47.3 ***
Living with partner	59.9	43.1
Child living with parents	2.2	4.4
Single parent	4.8	5.2
Low educational level %		
Immigrant status %	25.7	23.4
Relocated %	19.1	23.9 ***

*p < .05, ** *p *< .01, *** *p *< .0001

#### Functional Impairment

Participants completed the validated Dutch version of the RAND-36, which measures different concepts of functional status [18]. We examined functional status at T2 because functional status 18 months post-disaster is more stable than functional status three weeks after the disaster. The physical and mental health summary scales were calculated using the means scores of the US population. In order to produce easily interpretable odds ratios, we dichotomized the summary scales: cut-off scores were based on the mean score from a normal Dutch population minus one standard deviation [19,20].

#### Psychological Distress

Feelings of depression and anxiety were measured by the Dutch version of the SCL-90 [21,22], which assesses the degree of depression and anxiety during the past week. We dichotomized the scales into 'very high' and 'high' (80^th ^percentile) versus 'above average', 'average', and 'below average', according to established references for the healthy Dutch population [23].

*Symptoms Presented to the GP *– We used all information on symptoms and diagnoses that was registered in the EMRs of survivors from the day after the disaster until two years post-disaster (half a year after T2). Symptoms and diagnoses were registered by the GP in accordance with the ICPC [17].

To examine whether survivors presented their self-reported symptom to the GP, we compared self-reported symptoms with corresponding symptoms in the EMRs of survivors. For example, headache on the symptom-scale was compared with the ICPC codes N01 (headache) and N02 (tension headache). We compared the VOEG items with one to four corresponding ICPC codes, except for the item 'pain in bones and muscles', which we compared with 21 different ICPC codes (table 1).

To evaluate whether or not symptoms were medically unexplained, we used 'episodes of care' that were constructed by GPs. An episode of care is the period from the first presentation of a health problem to a health care provider until the completion of the last encounter for that same health problem [22]. Symptoms that were not associated with an ICPC diagnosis after clinical judgment or after medical examination at some point during the episode of care were defined as medically unexplained.

### Statistical analyses

Information from the health survey and the surveillance program was summarized and analyzed using SAS version 9.1. The percentages of survivors who presented symptoms to the GP were examined for various groups: survivors with no self-reported symptoms at T1 or T2 (no symptoms), survivors with self-reported symptoms at T1 but not at T2 (T1 only), survivors self-reported symptoms at T2 but not at T1 (T2 only), and survivors with self-reported symptoms at both T1 and T2 (persistent symptoms).

Adjusted odds ratios (OR) and 95% confidence intervals (CI) were calculated for the associations between medical care seeking for symptoms reported at T2 and functional impairment and distress reported at T2. We controlled for sex, age, educational level, and immigrant status.

Finally, we calculated the percentages of the symptoms that could not be related to a medical diagnoses in the episodes of care.

## Results

**Figure 1:**
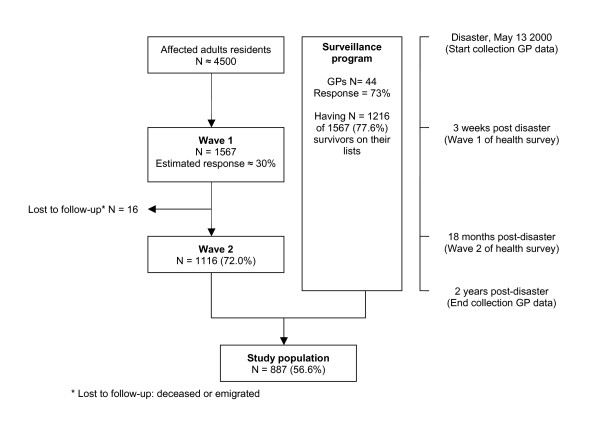
Flowchart of the study population.

The study population consisted of 887 survivors who participated at T1 and at T2 of the health survey, and who were registered in one of the participating general practices at the time of the disaster (figure 1). Survivors who participated at T1 and T2 of the health survey (N = 1116) were more likely to be female, were somewhat older, were more likely to live with a partner and had to relocate less often as a result of the disaster than survivors who did not participate in the health survey (N = 3285) (table 1). Among participants at both T1 and T2, survivors for whom the EMR was available (N = 887) were somewhat less likely to be employed than survivors for whom the EMR was not available (N = 229) (56.6% and 63.9% respectively, *p *< .05). Survivors for whom the EMR was available did not differ from those for whom the EMR was not available with regard to the other demographic characteristics (data not shown).

Table 2 shows the ICPC codes that correspond with the self-reported symptoms. Survivors were most likely to seek medical care for persistent self-reported symptoms. Except for pain in bones and muscles, about 25% of the persistent symptoms (T1 and T2) were also found in the medical records of survivors. Survivors with self-reported symptoms at only T2 were more likely to present their symptoms to the GP than survivors with self-reported symptoms at only T1. For example, survivors with stomachache at both T1 and T2 were five times more likely to seek medical care for their symptom than survivors without stomachache at either wave. In addition, survivors with stomachache at only T1 were 2.3 times, and those with stomachache at only T2 were 3.6 times more likely to visit the GP with stomachache compared to survivors who did not report this symptom in the symptom scale.

**Table 2:** Number of survivors who presented symptoms to the GP that correspond with self-reported symptoms*

**Symptoms on symptom-scale (corresponding ICPC codes)**	**Self-reported symptoms at T1 and/or T2**	**No. of Survivors with self-reported symptom (%)**	**No. of survivors who presented symptom to the GP (%)**†	**Risk ratio**‡
Fatigue	No symptoms	93 (11.4)	3 (3.2)	1.0
(ICPC codes: A04, P06)	T1 only	158 (19.3)	22 (13.9)	4.3
	T2 only	0 (0.0)	0 (0.0)	0
	T1 and T2	568 (69.4)	162 (28.5)	8.9
				
Pain in bones and muscles	No symptoms	278 (35.2)	127 (45.7)	1.0
(ICPC codes: L01 – L20, L29)	T1 only	85 (10.7)	46 (54.1)	1.2
	T2 only	163 (20.6)	104 (63.8)	1.4
	T1 and T2	265 (33.5)	169 (63.8)	1.4
				
Back pain	No symptoms	318 (40.5)	31 (9.8)	1.0
(ICPC codes: L02, L03)	T1 only	65 (8.3)	10 (15.4)	1.6
	T2 only	150 (19.1)	44 (29.3)	3.0
	T1 and T2	252 (32.1)	73 (29.0)	3.0
				
Stomachache	No symptoms	366 (41.3)	21 (5.7)	1.0
(ICPC codes: D01 – D03, D06)	T1 only	122 (13.8)	16 (13.1)	2.3
	T2 only	141 (15.8)	29 (20.6)	3.6
	T1 and T2	258 (29.1)	73 (28.3)	5.0
				
Headache	No symptoms	271 (34.2)	12 (4.4)	1.0
(ICPC codes: N01, N02)	T1 only	146 (18.4)	9 (6.2)	1.4
	T2 only	97 (12.3)	13 (13.4)	3.1
	T1 and T2	278 (35.1)	59 (21.2)	4.8
				
Shortness of breath	No symptoms	446 (56.2)	10 (2.2)	1.0
(ICPC codes: R02 – R04, R29)	T1 only	71 (9.0)	0 (0.0)	0
	T2 only	118 (14.9)	13 (11.0)	5
	T1 and T2	158 (19.9)	34 (21.5)	9.8
				
Dizziness	No symptoms	459 (58.2)	15 (3.3)	1.0
(ICPC code: N17)	T1 only	103 (13.0)	3 (2.9)	0.9
	T2 only	62 (7.9)	4 (6.5)	2.0
	T1 and T2	165 (20.9)	26 (15.8)	4.8
				
Pain in chest and region of heart	No symptoms	501 (64.1)	23 (4.6)	1.0
(ICPC codes: L04, K01 – K03)	T1 only	66 (8.5)	10 (15.2)	3.3
	T2 only	96 (12.3)	17 (17.7)	3.9
	T1 and T2	118 (15.1)	32 (27.1)	5.9

GP: general practitioner; ICPC: International Classification of Primary Care

* Due to missing values on T1 and T2, N-values differ from the total study population (N = 887);

† Survivors with a symptom (ICPC code) corresponding with the self-report symptom in their electronic medical record (EMR)

‡ Risk ratio: % of survivors with self-reported symptoms who presented symptoms to the general practitioner/% of survivors without self-reported symptoms who presented symptoms to the general practitioner

Table 3 shows the percentage of survivors who reported a high level of functional impairment, depression, anxiety or physical symptoms at T2. Percentages are compared between survivors who did or did not present self-reported symptoms at T2 to the GP in the two years after the disaster. Survivors who sought medical care for fatigue were more likely to have poor mental and physical health as reported on the RAND-36 summary scales and a high level of psychological distress and physical symptoms at T2 than survivors who did not present fatigue to the GP. In addition, survivors who presented pain in bones and muscles, pain in back and stomachache to the GP were somewhat more likely to report poor physical health on the RAND-36 summary scale and to report a high level of physical symptoms at T2. This pattern was not found for headache, shortness of breath, dizziness, or pain in chest and region of the heart.

**Table 3:** Functional status and distress among survivors who did and did not present self-reported symptoms at T2 to the GP

	**Presented self-reported symptom to GP (%)**	**Did not present self-reported symptom to GP (%)**	Adjusted OR (95% CI)*
Fatigue	N = 162	N = 406	
Poor physical health†	42.3	25.3	1.9 (1.2–3.1)
Poor mental health†	59.4	39.2	2.4 (1.5–3.7)
Feelings of depression (high)	61.0	44.3	2.1 (1.4–3.2)
Feelings of anxiety (high)	56.4	40.2	2.0 (1.3–3.0)
Physical symptoms (high)‡	81.5	65.3	2.1 (1.3–3.4)
			
Pain in bones and muscles	N = 273	N = 155	
Poor physical health†	46.7	19.2	3.5 (2.0–6.4)
Poor mental health†	44.1	37.6	1.3 (0.8–2.1)
Feelings of depression (high)	54.2	47.6	1.3 (0.8–2.1)
Feelings of anxiety (high)	49.0	41.3	1.3 (0.8–2.1)
Physical symptoms (high)‡	75.5	64.5	1.6 (1.0–2.7)
			
Back pain	N = 117	N = 285	
Poor physical health†	41.1	27.7	1.9 (1.1–3.3)
Poor mental health†	42.1	41.1	1.0 (0.6–1.7)
Feelings of depression (high)	48.6	51.5	0.8 (0.5–1.3)
Feelings of anxiety (high)	44.4	42.5	0.8 (0.5–1.3)
Physical symptoms (high)‡	75.2	69.8	1.4 (0.8–2.3)
			
Stomachache	N = 102	N = 297	
Poor physical health†	43.6	28.5	1.6 (0.8–2.9)
Poor mental health†	50.0	47.4	1.1 (0.6–1.9)
Feelings of depression (high)	57.8	53.7	1.0 (0.6–1.6)
Feelings of anxiety (high)	61.1	46.2	1.6 (0.9–2.7)
Physical symptoms (high)‡	86.3	77.1	1.8 (0.9–3.6)
			
Headache	N = 72	N = 303	
Poor physical health†	36.4	29.1	0.9 (0.4–1.8)
Poor mental health†	58.2	47.3	1.2 (0.6–2.3)
Feelings of depression (high)	56.9	51.2	0.9 (0.5–1.7)
Feelings of anxiety (high)	52.9	51.9	0.8 (0.4–1.4)
Physical symptoms (high)‡	81.9	75.9	1.0 (0.5–2.0)
			
Shortness of breath	N = 47	N = 229	
Poor physical health†	48.4	41.1	0.8 (0.3–2.1)
Poor mental health†	48.4	52.0	0.6 (0.2–1.5)
Feelings of depression (high)	56.1	60.0	0.6 (0.3–1.5)
Feelings of anxiety (high)	54.8	54.2	0.8 (0.4–1.8)
Physical symptoms (high)‡	70.2	82.1	0.4 (0.2–0.9)
			
Dizziness	N = 30	N = 197	
Poor physical health†	52.9	44.3	1.1 (0.4–3.5)
Poor mental health†	52.9	55.0	0.9 (0.3–2.8)
Feelings of depression (high)	53.9	64.5	0.7 (0.3–1.7)
Feelings of anxiety (high)	67.9	60.4	1.5 (0.5–3.8)
Physical symptoms (high)‡	93.3	87.8	1.5 (0.3–7.6)
			
Pain in chest and region of heart	N = 49	N = 165	
Poor physical health†	37.5	39.5	0.6 (0.2–1.4)
Poor mental health†	68.8	58.9	1.5 (0.6–3.8)
Feelings of depression (high)	64.3	62.9	0.8 (0.3–1.7)
Feelings of anxiety (high)	73.3	60.8	1.2 (0.5–2.7)
Physical symptoms (high)‡	87.8	82.4	1.3 (0.5–3.6)

GP = general practitioner

* Adjusted for possible confounders, sex, age, educational level and ethnicity

† A low score on the physical and mental component score of the RAND-36.

‡ Six or more symptoms on the 13-item symptoms scale, which is one standard deviation above the reference mean.

Table 4 gives the number of survivors who presented their self-reported symptom to the GP and the number of symptoms presented to the GP, demonstrating that many survivors presented the same symptom several times to the GP in the two years post-disaster. The majority of symptoms were not related to a diagnosis in an episode of care, and were defined as MUS. Fatigue was most often unexplained (90.9%), followed by headache (85.6%). Shortness of breath and pain in the chest and the region of the heart were least often defined as MUS (both 55.8%).

**Table 4:** Diagnoses most frequently associated with symptoms presented to the GP and percentage of symptoms labeled as MUS

**Self-reported symptom**	**No. of survivors that presented self-reported symptom to the GP**	**No. of symptoms presented to GP**	**Diagnosis after clinical judgment of the GP**	**%**
Fatigue	180	420	Various diagnoses	9.1
			*No diagnosis/MUS*	*90.9*
				
Pain in bones and muscles	319	1102	Musculoskeletal disease, other	3.5
			Back syndrome with radiating pain	3.0
			Various diagnoses	21.8
			*No diagnosis/MUS*	*71.7*
				
Back pain	127	268	Back syndrome with radiating pain	13.4
			Arthrosis spinal column	5.6
			Various diagnoses	5.6
			*No diagnosis/MUS*	*75.4*
				
Stomachache	118	317	Oesophagus disease	11.0
			Peptic ulcer other	5.4
			Irritable bowel syndrome	3.2
			Stomach function disorder	3.2
			Various diagnoses	13.2
			*No diagnosis/MUS*	*64.0*
				
Headache	81	139	*Migraine*	*7.2*
			*Various diagnoses*	*7.2*
			*No diagnosis/MUS*	*85.6*
				
Shortness of breath	47	95	Asthma	14.7
			Acute bronchitis	10.5
			Various diagnoses	18.8
			*No diagnosis/MUS*	*55.8*
				
Dizziness	33	46	Vertiginous syndrome	10.9
			*No diagnosis/MUS*	*89.1*
				
Pain in chest and region of the heart	59	95	Various diagnoses	44.2
			*No diagnosis/MUS*	*55.8*

GP = general practitioner; MUS = medically unexplained symptoms

## Discussion and conclusion

The majority of symptoms reported by survivors of the fireworks disaster was not presented to the GP. Survivors with persistent symptoms (symptoms reported at both T1 and T2) were more likely to seek medical care for their symptoms than survivors with self-reported symptoms at only one timepoint. Presenting the individual self-reported symptoms to the GP was not consistently associated with a high level of functional impairment and distress. When presented to the GP, the majority of symptoms could not be related to a medical disorder in the episode of care.

To our knowledge, this is the first study that has compared self-reported symptoms among survivors of a disaster with the symptoms that were registered in the medical records. Despite this, some potential limitations of the present study deserve attention. Firstly, selective response and possible bias is of concern in this study. Shortly after the disaster, all affected residents were registered; this database was used to detect possible demographic differences between participants and non-participants at T1. Only an estimated 30% of all affected residents participated in the questionnaire health survey, and the EMRs were not available for all survivors who participated in the health survey. Compared to the total affected group, the study population in the present study (participants at both T1 and T2) were more likely to be women, living with a partner, aged between 45 and 64 years and were more often relocated as a result of the disaster. In addition, participants at both T1 and T2 for whom the EMR was available were less likely to have a paid job than participants at both T1 and T2 for whom the EMR was not available. In two previous studies, we examined whether selective response resulted in biased prevalence estimates of health problems among survivors [14,23]. In these studies, multiple imputation, a statistical technique that uses a statistical model to fill in plausible values for each missing data point, was used [24,25]. Because the missing values are drawn from a distribution, a range of values is imputed for each missing value (e.g. five values), with variation appropriately reflecting the uncertainty about that value. Using this technique, it can be estimated what the prevalence of the outcomes of interest would have been if there had been no (systematic) attrition in the longitudinal study. The results from multiple imputation indicated that, despite selective response, the imputed prevalence estimates did not significantly differ from the crude prevalence estimates of health problems, indicating that the selective response did not result in highly biased prevalence estimates.

Secondly, we compared self-reported symptoms with ICPC codes registered in the EMRs of survivors [17]. Therefore, we have to consider the sensitivity and specificity of the ICPC codes corresponding to the self-reported symptoms. The GP might not register all symptoms that the patient presented. There may be some false negatives, and the percentage of survivors presenting self-reported symptoms may be an underestimation of the actual number of symptoms that are presented to the GP. In addition, table 2 shows percentages of survivors who had no self-reported symptoms at T1 or T2 but for whom symptoms were registered in the EMRs. These cases are not necessarily false positives, since the symptoms could have been correctly registered, if the symptoms occurred only between the two waves.

Compared to self-rating scales, relatively little is known about the validity and reliability of GP records. Despite this, a high level of agreement (mean 81%) between 161 GPs with regard to ICPC coding was found in the second Dutch national survey of general practice. This study indicated that health problems registered by GPs according to the ICPC provide a reliable overview of morbidity [26]. Other studies have also shown that ICPC coding is a valid method for studying morbidity [27]. In addition, the ICPC allows registration of symptoms with a high level of specificity, since the symptoms and complaints can be registered at the symptom level in the EMRs of patients. This is an important advantage compared to other classification systems. Despite this, it is possible that the GP did not register all symptoms presented by a patient. Indeed, most survivors have multiple symptoms and it is likely that the survivor presents, or the GP registers, only the most important symptom(s). This could have resulted in an underestimation of the symptoms presented to the GP.

Thirdly, we could not compare the self-report data with GP data for a control group. Despite this, our findings are comparable to the findings from general population studies in which only a minority of symptoms was presented to the GP [2,3]. For example, Green et al. have shown that in the US general population, adults visit a physician for about 25% of the symptoms they experience [2].

Since causal attributions and illness perceptions are strongly related to health care utilization for symptoms, it is possible that survivors are more likely to seek medical care for symptoms when they attribute their symptoms to exposure to toxic substances from a disaster [28,29]. Following an aircraft disaster in Amsterdam, survivors attributed their symptoms to depleted uranium. After this disaster, 53 to 80% of reported symptoms were known to the GP [30]. We have reason to believe that the survivors of the Enschede fireworks disaster did not attribute their symptoms to exposure to toxic substances. Three weeks after the disaster blood and urine samples were taken to examine possible exposure to toxic substances [12]. The results did not indicate elevated body burden. In the aftermath of the fireworks disaster no conspiracy theories about health problems due to exposure to toxic substances developed

To date, most studies that have examined MUS have been performed in primary or secondary care and have examined symptoms for which patients sought medical attention. The present study examined whether self-reported symptoms among survivors of a man-made disaster were presented to the GP, and showed that the majority of self-reported symptoms was not presented to the GP. Symptoms that were reported at only T1 were less often presented to the GP than symptoms reported at only T2 or persistent symptoms (at both T1 and T2). It is possible that survivors were mostly impaired by psychological problems such as anxiety and depression shortly after the disaster, and that they only sought medical help for physical symptoms after a longer period of time, when these symptoms became persistent or disabling. It can also be speculated that symptoms at T1 were likely to be transient or were explained by the survivors as a normal reaction to the disaster. Indeed, cognitions about symptoms affect medical care-seeking decisions. For example, Sensky et al. found that frequent attenders of general practice had fewer normalizing explanations for their symptoms than the comparison group [28]. In addition, Cameron et al., found that symptoms attributed to stress rather than to illness were less likely to be presented to the GP [31]. Although survivors presented only a minority of their symptoms to the GP, it is important to note that most survivors have multiple symptoms. In a previous study we found that 33% of the survivors of the fireworks disaster reported 10 or more symptoms in the health survey [32]; it is unlikely that survivors present all these symptoms to the GP. Instead, it is more likely that survivors report only the most important or worrisome symptoms to the GP. Since the number of symptoms is strongly related to the degree of functional impairment [5,33], it is important that GPs ask patients about additional symptoms.

In a recent study among survivors of the fireworks disaster, the authors found that self-reported symptoms were strongly related to a high level of functional impairment and psychological problems [32]. For that reason, we examined whether survivors with a high level of functional impairment and psychological distress were more likely to seek medical care for their self-reported symptoms. Survivors who presented fatigue to the GP were significantly more likely to have a high level of impairment and distress. Survivors who presented pain in bones and muscles, pain in back and stomachache to the GP were more likely to report a poor physical health and a have a high level of physical symptoms at T2 compared to those who did not present these symptoms to the GP. This pattern was, however, not found for the other symptoms. Apparently, a high level of impairment and distress was not the major reason to seek medical care for individual symptoms. It is likely that the decision to consult a GP was based on other factors, such as perceived susceptibility to illness, perceived severity of the symptom or beliefs about the cause of the symptoms [28,29,31,33].

Of the self-reported symptoms that were also presented to the GP, 56% to 91% remained medically unexplained in the episode of care. This finding is consistent with a study among survivors of an airplane crash in Amsterdam in which it was shown that 57% to 85% of symptoms presented to the GP remained unexplained [30]. General population studies have found similar percentages [1,4]. The studies following disasters suggest that, as in the general population, physical symptoms such as headache, stomachache and fatigue among survivors are likely to remain medically unexplained after clinical judgment of a physician.

In conclusion, the majority of self-reported symptoms among the survivors of the fireworks disaster were not presented to the GP. On the one hand, this indicates that some symptoms reported in epidemiologic studies after traumatic events are transient and not a reason to seek medical care. On the other hand, this study shows that the symptoms presented to the GP are only the tip of the iceberg of symptoms that are related to functional impairment among survivors. The results of the present study indicate that the survivors who present their symptoms to the GP are not always those who have a high level of functional impairment and distress. When presented to the GP, most symptoms could not be related to a medical disorder, and were labeled as MUS. This result suggest that in a post-disaster context, just as in routine clinical practice, most physical symptoms reported on a symptom questionnaire will be transient, medically unexplained, or both.

## Abbreviations

GP – general practitioner

MUS – medically unexplained symptoms

EMR – electronic medical record

ICPC – International Classification of Primary Care

RAND-36 – Questionnaire on general health (36 items)

SCL-90 – Symptom Checklist (90 items)

VOEG – Questionnaire into Subjective Health Complaints

OR – odds ratio

CI – confidence interval

## Competing interests

The author(s) declare that they have no competing interests.

## Authors' contributions

BvdB analyzed the data and was the principal author of the paper. LG contributed to the interpretation of the data and the writing of the paper. RKS was involved in the statistical analysis and the interpretation of the data. CJY, PvdV and EL critically reviewed the manuscript. CJY, RKS, PvdV, EL and LG were involved in the acquisition of the data, conception, and design of the study.

## Pre-publication history

The pre-publication history for this paper can be accessed here:


